# Prognostic significance of tumoral Siglec-15 expression in solid tumors: a systematic review and meta-analysis

**DOI:** 10.3389/fimmu.2026.1905631

**Published:** 2026-07-21

**Authors:** Ningning Zhang, Yuan He, Zemin Qin, Junyan Yu, Wei Guo

**Affiliations:** 1 Department of Oncology, Heping Hospital Affiliated to Changzhi Medical College, Changzhi, Shanxi, China; 2 Department of Gastrointestinal Surgery, Heping Hospital Affiliated to Changzhi Medical College, Changzhi, Shanxi, China

**Keywords:** immune checkpoint, meta-analysis, overall survival, prognosis, Siglec-15, solid tumors

## Abstract

**Background:**

Siglec-15 has emerged as a novel immune checkpoint molecule and a potential therapeutic target in solid tumors. Previous studies reported inconsistent prognostic significance of tumoral Siglec-15 expression across cancer types. We therefore conducted a systematic review and meta-analysis to comprehensively evaluate the association of tumoral Siglec-15 expression with survival outcomes in patients with solid tumors.

**Methods:**

PubMed, Web of Science, and EMBASE were systematically searched from database inception to March 30, 2026. Observational studies investigating Siglec-15 protein expression in tumor cells and prognosis were included. Study quality was assessed using the Newcastle-Ottawa Scale. Random-effects models were applied to calculate pooled hazard ratios (HRs) and odds ratios (ORs) with corresponding 95% confidence intervals (CIs). Prespecified subgroup analyses, meta-regression analyses, and publication bias assessment were performed.

**Results:**

Twenty-seven datasets involving 4075 patients were included. High tumoral Siglec-15 expression was significantly associated with poorer overall survival (OS, HR = 1.53, 95% CI: 1.25-1.86, *P* < 0.001) and worse disease-free/recurrence-free survival (DFS/RFS, HR = 1.30, 95% CI: 1.09-1.55, *P* = 0.004). No significant association was observed for progression-free survival (HR = 1.03, 95% CI: 0.67-1.56, *P* = 0.903). High Siglec-15 expression showed a marginal association with improved disease-specific survival (DSS, HR = 0.66, 95% CI: 0.43-1.00, *P* = 0.050). Tumor-specific analyses demonstrated significantly worse OS in colorectal cancer, gastric cancer, and osteosarcoma, whereas breast cancer showed a non-significant association in the opposite direction (HR = 0.72, 95% CI: 0.33-1.56). Tumoral Siglec-15 expression was inversely associated with PD-L1 positivity (OR = 0.40, 95% CI: 0.23-0.72, *P* = 0.002), supporting a mutually exclusive immune checkpoint pattern. No significant associations were observed between Siglec-15 expression and most clinicopathological features.

**Conclusions:**

Elevated tumoral Siglec-15 expression was associated with unfavorable OS and DFS/RFS, whereas the borderline association with improved DSS should be interpreted cautiously because it was based on only three studies. These findings support a highly cancer-type dependent role of Siglec-15 as a clinically relevant prognostic biomarker and a promising immunotherapeutic target. Further prospective studies are needed to validate these findings and develop standardized assessment strategies, preferably using fixed cutoff values and simplified scoring methods.

## Introduction

Immune checkpoint inhibitors (ICIs) targeting the programmed death-1 (PD-1)/programmed death ligand-1 (PD-L1) axis have transformed the landscape of many malignancies. Durable clinical responses and prolonged survival have been achieved in several tumor types, including melanoma, non-small cell lung cancer (NSCLC), renal cell carcinoma, and urothelial carcinoma (1–3). Despite these advances, only a subset of patients derives meaningful benefit from PD-1/PD-L1 blockade. Objective response rates generally remain low below 30% in unselected populations, and both primary and acquired resistance continue to limit long-term efficacy (4–7). Moreover, patients with PD-L-negative tumors often have limited opportunities to benefit from currently available immunotherapeutic strategies. These challenges have prompted increasing efforts to identify alternative immune checkpoints that contribute to tumor immune evasion (8, 9).

Sialic acid-binding immunoglobulin-like lectin 15 (Siglec-15) has emerged as a promising candidate in this context. Siglec-15 is a type I transmembrane protein belonging to the Siglec family and shares structural similarities with members of the B7 immune regulatory family (10, 11). Under physiological conditions, its expression is largely restricted to osteoclasts and selected myeloid cell populations (12). In contrast, aberrant expression of Siglec-15 has been observed in a wide range of human cancers, including colorectal, gastric, lung, pancreatic, and breast cancers (11, 13). Experimental studies have shown that Siglec-15 promotes immune escape by suppressing T-cell activation and proliferation while simultaneously supporting the development of an immunosuppressive tumor microenvironment through macrophage-mediated mechanisms (14–16). Beyond its immunoregulatory functions, increasing experimental evidence indicates that Siglec-15 also acts as a tumor-cell-intrinsic regulator of malignant progression. Independent studies have demonstrated that Siglec-15 promotes tumor-cell proliferation, migration, invasion, epithelial-mesenchymal transition, and resistance to apoptosis through activation of multiple oncogenic signaling pathways, including PI3K/Akt, MAPK, STAT3, and JAK2/STAT3 (17–19). These findings suggest that Siglec-15 should be regarded not only as a non-classical immune checkpoint molecule but also as a direct driver of tumor progression, thereby providing an additional biological rationale for its prognostic significance across different solid tumors.

Although Siglec-15 shares immunosuppressive properties with established immune checkpoint molecules, accumulating evidence suggests that its biological regulation differs fundamentally from that of the PD-1/PD-L1 axis (11). This distinction has attracted considerable interest because it may explain why a substantial proportion of patients fail to respond to conventional PD-1/PD-L1 blockade despite exhibiting immunologically active tumors. An important feature distinguishing Siglec-15 from PD-L1 is its largely non-overlapping expression pattern. Previous studies have demonstrated that Siglec-15 expression is frequently enriched in tumors with low or absent PD-L1 expression, suggesting that it may represent an alternative immune suppressive pathway independent of the PD-1/PD-L1 axis (11, 20). This biological characteristic has generated considerable interest in Siglec-15-directed therapies, particularly for patients who are unlikely to respond to conventional checkpoint blockade. Several anti-Siglec-15 antibodies have progressed through early-phase clinical evaluation. NC318 has demonstrated a manageable safety profile and showed a disease control rate of 34.9% in advanced or metastatic solid tumors (NCT03665285). Another antibody PYX-106 is evaluated in an ongoing phase I study for tolerability and pharmacodynamic activity (NCT05718557). Collectively, these studies support the continued clinical development of Siglec-15-directed immunotherapy while highlighting the need for predictive biomarkers to identify patients most likely to benefit from treatment.

Over the past few years, numerous clinical studies have evaluated the prognostic value of tumoral Siglec-15 expression. However, the findings remain inconsistent. While several investigations reported associations between elevated Siglec-15 expression and poor survival outcomes (17, 21, 22), others observed no significant prognostic effect or even suggested favorable outcomes in specific tumor types (23–27). Differences in tumor biology, patient populations, immunohistochemical (IHC) assessment methods, and cutoff value definitions may partially account for these discrepancies. A previous meta-analysis synthesized evidence from 13 studies involving 1376 patients and suggested that elevated Siglec-15 expression was associated with unfavorable prognosis in solid tumors (28). However, the number of available studies included in this meta-analysis was relatively small, precluding comprehensive subgroup analyses according to cancer type, scoring method, cutoff definition, and data source.

Since more studies across multiple tumor types have being recently published, an updated and more comprehensive synthesis is required to provide a more robust assessment of the prognostic significance and clinical implications of tumoral Siglec-15 expression. We hypothesized that the prognostic value of tumoral Siglec-15 expression is highly cancer-type dependent and inversely linked with PD-L1 status. Therefore, we conducted this systematic review and meta-analysis to investigate the association between tumoral Siglec-15 expression and survival outcomes, as well as clinicopathological characteristics, in patients with solid tumors.

## Materials and methods

### Literature search strategy

This systematic review and meta-analysis was conducted in accordance with the Preferred Reporting Items for Systematic Reviews and Meta-Analyses (PRISMA) guidelines (29). A comprehensive literature search of PubMed, Web of Science, and EMBASE databases from inception to March 30, 2026, was performed to identify studies investigating the associations between Siglec-15 protein expression in tumor cells and survivals outcomes in patients with solid tumors. MeSH terms and relevant free-text keywords were used as follows: (“Siglec-15”[Title/Abstract] OR “siglec15”[Title/Abstract] OR “sialic acid-binding immunoglobulin-like lectin 15”[Title/Abstract]) AND (“Neoplasms”[Mesh] OR cancer[Title/Abstract] OR tumor[Title/Abstract] OR tumor[Title/Abstract] OR carcinoma[Title/Abstract] OR neoplasm[Title/Abstract] OR malignancy[Title/Abstract]). The complete search strategies for all databases are provided in **Supplementary Table 1**. Only studies published in English were included. In addition, the reference lists of eligible articles were manually screened to identify additional relevant studies.

### Inclusion and exclusion criteria

Eligibility of studies was assessed according to the PECOS framework:

P (Participants): Patients with histologically confirmed solid tumors.

E (Exposure): High expression of Siglec-15 protein in tumor cells.

C (Comparator): Low or negative expression of Siglec-15 protein in tumor cells.

O (Outcomes): Survival outcomes and clinicopathological characteristics.

S (Study design): Observational cohort studies.

The primary outcome was overall survival (OS). Second outcomes included progression-free survival (PFS), recurrence-free survival (RFS), disease-free survival (DFS), disease-specific survival (DSS), and clinicopathological parameters, including gender, age, tumor differentiation, tumor size, lymph node metastasis (LNM), distant metastasis, TNM stage, and PD-L1 expression in tumor cells.

The exclusion criteria were as follows: (1) studies including patients with hematological malignancies; (2) studies evaluating prognostic value based solely on public databases without independent tissue cohorts; (3) studies assessing Siglec-15 expression at the transcriptional level; (4) studies measuring Siglec-15 in serum, stromal cells, or tumor-associated macrophages (TAMs); (5) studies not reporting hazard ratios (HRs) or lacking Kaplan-Meier curves for data extraction; (6) studies without available full text. Two investigators (YH and ZQ) independently performed study selection, discrepancies were resolved through discussion with a third reviewer (NZ).

### Data extraction

Data from each eligible study were independently extracted by two investigators (YH and ZQ), including first author, publication year, country, tumor type, sample size, detection method of Siglec-15 protein expression, IHC scoring system, cutoff values and their determination methods, survival outcomes, and clinicopathological features. When both univariable and multivariable HRs with corresponding 95% confidence intervals (CIs) were reported, multivariable estimates were preferentially extracted because they better accounted for potential confounding factors. When HRs and corresponding 95% CIs were not directly reported, survival data were extracted from Kaplan-Meier curves using Engauge Digitizer software by two independent investigators (YH and ZQ), and HRs and 95% CIs were subsequently estimated. Agreement of HR values between the two independent extractors was assessed using the intraclass correlation coefficient (ICC) based on a two-way mixed-effects model. In present study, the inter-rater agreement between the two extractors was excellent (ICC = 0.983, 95% CI: 0.958-0.994; **Supplementary Table 2**), indicating high reproducibility of the extracted survival data. Any discrepancies were resolved through discussion with a third reviewer (NZ).

### Assessment of study quality

Study quality was independently evaluated by two investigators (YH and ZQ) using the Newcastle-Ottawa Scale (NOS) for cohort studies (30). The NOS evaluates studies based on selection, comparability, and outcome domains, with a maximum score of nine stars. Studies scoring 7–9 were considered high quality, 4–6 moderate quality, and < 4 low quality. Disagreements were resolved by consensus with a third reviewer (NZ).

### Statistical analysis

All statistical analyses were performed using STATA v18.0 (StataCorp, TX, US). Between-study heterogeneity was assessed using the *I*^2^ statistic, with *I*^2^ < 25%, 25-50%, and > 50% indicating low, moderate, and high heterogeneity, respectively. These thresholds were used descriptively to characterize heterogeneity rather than to determine model selection. Because considerable clinical and methodological diversity across studies, random-effects models were prespecified for all pooled analyses regardless of the observed *I*^2^ value. Pooled HRs and 95% CIs were calculated to evaluate associations between Siglec-15 expression and survival outcomes, while pooled odds ratios (ORs) and 95% CIs were used for clinicopathological features. Subgroup analyses were conducted according to country (China *vs*. outside China), sample size (< 100 *vs*. ≥ 100), IHC scoring method (immunoreactive score [IRS]/H-score *vs*. count/percentage/intensity only), cutoff determination method (median *vs*. fixed *vs*. optimal value), HR type (univariate *vs*. multivariate), data source (reported *vs*. estimated), study quality (high quality *vs*. moderate/low), and cancer type. Between-subgroup difference was analyzed by meta-regression when ≥ 10 studies were available. Accordingly, univariate meta-regression analyses were used to explore the potential influence of baseline clinicopathological characteristics, including total sample size, percentage of male patients, percentage of TNM III/IV stage tumors, percentage of patients with LNM, percentage of poorly differentiated tumors, on survival outcomes. Sensitivity analysis was conducted to assess the robustness of pooled results. Publication bias was assessed by viewing the symmetry of funnel plots and Egger’s test when ≥ 10 studies were included. A two-sided *P* value < 0.05 was considered statistically significant.

## Results

### Baseline characteristics of studies included in meta-analysis

**Figure 1:**
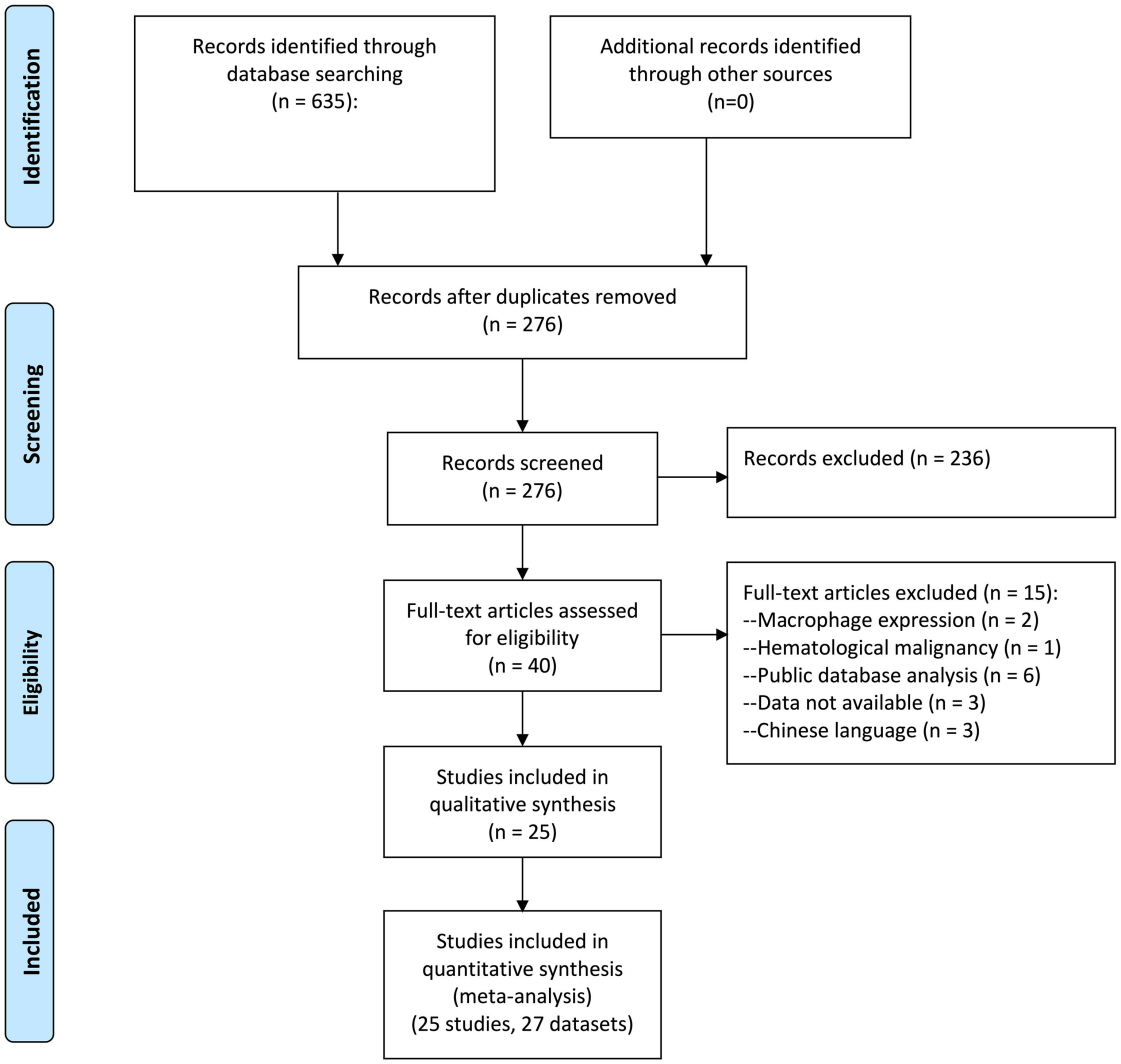
Flow diagram of literature selection.

A total of 276 unique articles were identified through database searching and removal of duplicates (**Figure 1**). After screening titles and abstracts, 236 irrelevant articles were excluded. Full-text assessment further excluded 15 studies (**Supplementary Table 3**). Ultimately, 25 studies were included in the meta-analysis (17–19, 21–23, 25–27, 31–46). One study reported three independent tumor datasets (40). Therefore, a total of 27 datasets comprising 4075 patients were included in the quantitative synthesis (**Table 1**).

**Table 1:** Baseline characteristics of studies included in the meta-analysis.

Study	Country	Tumor	Detection method	Scoring method	Cutoff determination	No. of high/low expression	Survival data source	HR type	Survival outcomes	NOS
Chen X, 2022 (23)	China	PDAC	IHC	Percentage	Optimal (≥ 5%)	49/214	Reported	Multivariate	PFS, DSS	7
Chen X, 2024 (31)	China	LSCC	IHC	NR	NR	61/50	Reported	Multivariate	OS	8
Chen Z, 2024 (31)	China	HCC	IHC	IRS	Median	117/104	Reported	Univariate	OS, DFS	8
Chu C, 2020 (34)	China	peSCC	IHC	Count	≥ 1	109/61	Reported	Univariate	DSS	7
Chu C, 2025	China	Bladder cancer	IHC	IRS	≥ 1	58/11	Reported	Univariate	OS, DSS	9
Cozac-Szoke 2025 (21)	Romania	Gastric cancer	IHC	H-score	Median (≥ 110)	57/76	Reported	Univariate	OS	6
Cui L, 2022 (35)	China	RLPS	IHC	IRS	≥ 5	52/39	Reported	Univariate	OS, DFS	8
Fan M, 2021 (17)	China	Osteosarcoma	IHC	IRS	≥ 4	16/20	Estimated	Univariate	OS	8
Hou X, 2022 (18)	China	Thyroid cancer	IHC	IRS	Median	44/42	Estimated	Univariate	OS	6
Jin T, 2023 (36)	China	PTC	IHC	IRS	Median (≥ 1)	92/152	Reported	Multivariate	PFS	7
Lai H, 2024 (25)	China	Breast cancer	IHC	IRS	Optimal (≥ 5)	54/20	Reported	Multivariate	OS	6
Li B, 2020 (37)	China	LUAD	IHC	Count	≥ 1	17/86	Reported	Univariate	OS, PFS	9
Li T, 2022 (38)	China	PDAC	MF-IHC	NR	NR	119/90	Estimated	Univariate	OS, DFS	6
Liang G, 2025 (22)	China	CRC	IHC	IRS	≥ 5	32/58	Reported	Multivariate	OS	6
Lu Z, 2023 (39)	China	CRC	IHC	Staining intensity	≥ weak staining	389/416	Estimated	Univariate	OS, DFS	8
Quirino M, 2021 (26)	Brazil	Gastric cancer	IHC	IRS	≥ 1	53/18	Estimated	Univariate	OS, RFS	7
Shafi S, 2022 (YTMA423) (40)	US	NSCLC	QIF	QIF score	Median	122/122	Estimated	Univariate	OS, PFS	8
Shafi S, 2022 (YTMA465) (40)	US	HNSCC	QIF	QIF score	Median	21/21	Estimated	Univariate	OS, PFS	8
Shafi S, 2022 (YTMA489) (40)	US	Breast cancer	QIF	QIF score	Median	119/119	Estimated	Univariate	OS, PFS	8
Song K, 2022 (19)	China	Osteosarcoma	IHC	Percentage	≥ 1%	16/36	Estimated	Univariate	OS	8
Sun H, 2024 (41)	China	LUAD	IHC	IRS	≥ 4	30/63	Reported	Multivariate	OS	7
Tan Z, 2025	China	TNBC	IHC	NR	NR	31/33	Estimated	Univariate	OS	6
Wang J, 2023 (42)	China	Gliomas	IHC	IRS	≥ 5	50/42	Reported	Multivariate	OS	7
Yang W, 2021 (44)	China	ccRCC	IHC	IRS	Median	73/77	Estimated	Univariate	OS	7
Yang W, 2025 (43)	China	HNSCC	IHC	H-score	> 50	21/19	Estimated	Univariate	PFS	7
Zhan W, 2023 (45)	China	Colon adenocarcinoma	IHC	H-score	Median (≥ 120)	67/35	Reported	Univariate	OS	6
Zhao J, 2022 (46)	China	NPC	IF	NR	NR	69/113	Reported	Multivariate	OS	8

ccRCC, clear cell renal cell carcinoma; CRC, colorectal cancer; DFS, disease-free survival; DSS, disease-specific survival; HCC, hepatocellular carcinoma; HNSCC, head and neck squamous cell carcinoma; HR, hazard ratio; IF, immunofluorescence; IHC, immunohistochemistry; IRS, immunoreactive score; LSCC, laryngeal squamous cell carcinoma; LUAD, lung adenocarcinoma; MF-IHC, multiplexed fluorescence IHC; NOS, Newcastle-Ottawa Scale; NPC, nasopharyngeal carcinoma; NR, not reported; NSCLC, non-small cell lung cancer; OS, overall survival; PDAC, pancreatic ductal adenocarcinoma; peSCC, penile squamous cell carcinoma; PFS, progression-free survival; PTC, papillary thyroid carcinoma; QIF, quantitative immunofluorescence; RFS, recurrence-free survival; RLPS, retroperitoneal liposarcoma; TNBC, triple-negative breast cancer.

Most studies were conducted in China (n = 22), followed by the United States (n = 3), Romania (n =1), and Brazil (n =1). Tumor types included CRC (3 studies, 997 patients), lung cancer (3 studies, 440 patients), breast cancer (3 studies, 376 patients), PDAC (2 studies, 472 patients), thyroid cancer (2 studies, 330 patients), gastric cancer (2 studies, 204 patients), osteosarcoma (2 studies, 88 patients), head and neck squamous cell carcinoma (HNSCC, 2 studies, 82 patients), hepatocellular carcinoma (HCC, 1 study, 221 patients), nasopharyngeal carcinoma (NPC, 1 study, 182 patients), penile squamous cell carcinoma (peSCC, 1 study, 170 patients), clear cell renal cell carcinoma (ccRCC, 1 study, 150 patients), laryngeal squamous cell carcinoma (LSCC, 1 study, 111 patients), gliomas (1 study, 92 patients), retroperitoneal liposarcoma (RLPS, 1 study, 91 patients), and bladder cancer (1 study, 69 patients).

All studies applied IHC to assess Siglec-15 protein expression, except four that employed immunofluorescence (40, 46). Regarding scoring system, 15 studies used IRS or H-score incorporating both staining intensity and proportion of positive cells, 8 used either percentage/count or intensity alone, and 4 did not report scoring methods. For cutoff definitions, 9 studies used median values, 12 used fixed thresholds, and 2 applied optimal cutoffs determined by the X-tile method. Based on these criteria, 1938 patients were classified into the high-expression group and 2137 into the low-expression group.

Of the 27 independent datasets included in the meta-analysis, 23 provided OS data, 7 assessed PFS, 5 reported DFS/RFS, and 3 reported DSS. In addition, 25 datasets involving 3601 patients were included in the analysis of clinicopathological features (**Supplementary Table 4**). According to NOS assessment, study scores ranged from 6 to 9 (**Table 1**; **Supplementary Table 5**). Overall, 19 studies were classified as high quality and 7 as moderate quality.

### OS

**Figure 2:**
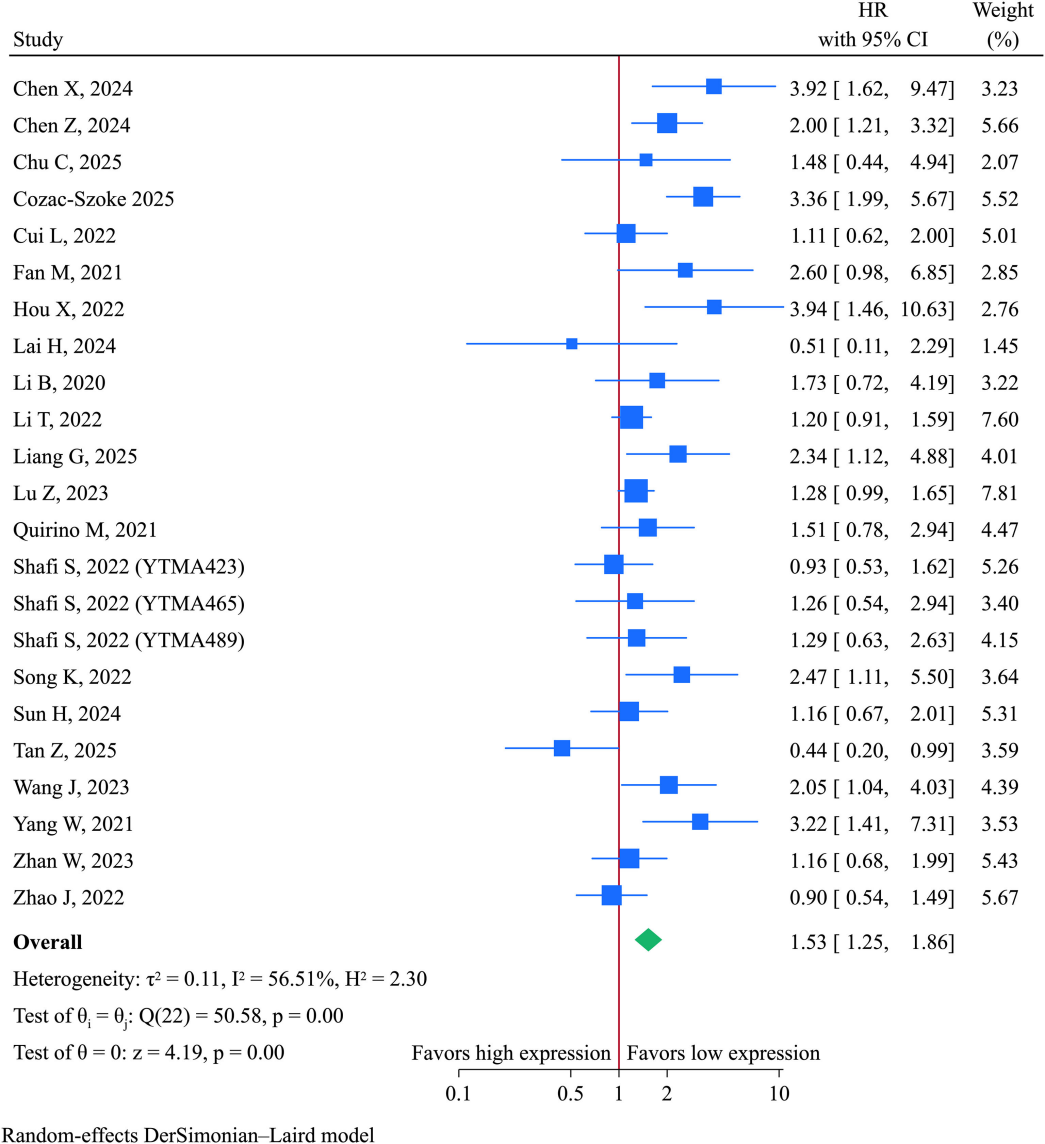
Forest plot of the association between tumoral Siglec-15 expression and OS in solid tumors. HR, hazard ratio; OS, overall survival.

A total of 23 datasets involving 1667 patients in the high-expression group and 1691 in the low-expression group were included. High heterogeneity was observed (*I*^2^ = 56.5%). Pooled analysis showed that high Siglec-15 expression was significantly associated with worse OS in solid tumors (HR = 1.53, 95% CI: 1.25-1.86, *P* < 0.001, **Figure 2**).

Subgroup analyses confirmed the robustness of this association across multiple stratifications, including country, sample size, HR type, data source, scoring method, cutoff definition, and study quality (**Table 2**; **Supplementary Figures 1**–**7**). Meta-regression analyses showed no significant influence of country (*P* = 0.944), sample size (*P* = 0.837), cutoff definition (*P* = 0.478), or study quality (*P* = 0.869) on pooled OS estimates. Specifically, separate pooled analyses demonstrated comparable effect estimates for studies reporting multivariable and univariable HRs (1.54 *vs.* 1.53, *P* = 0.999), indicating that adjustment status had minimal influence on the pooled association. The pooled effect sizes for reported and estimated HRs were also comparable (1.63 *vs.* 1.42, *P* = 0.542). Notably, no heterogeneity was observed in the subgroup using count/percentage/intensity scoring (*I*^2^ = 0%, n = 6) or in studies using fixed cutoff values (*I*^2^ = 0%, n = 10). These findings suggest that methodological differences in IHC scoring systems and cutoff definitions represent the primary source of between-study heterogeneity across solid tumors.

**Table 2:** Subgroup analysis of the association between Siglec-15 expression and overall survival.

Outcomes	No. of studies	No. of high/low expression	*I*^2^ (%)	HR (95% CI)	*P* for effect size	*P* for subgroup difference
Country						0.944
China	18	1295/1335	54.9	1.52 (1.22-1.89)	< 0.001	
Outside China	5	372/356	66.8	1.54 (0.93-2.54)	0.094	
Sample size						0.837
< 100	13	457/403	48.4	1.49 (1.08-2.06)	0.014	
≥ 100	11	1210/1288	65.7	1.55 (1.19-2.02)	0.001	
HR type						0.999
Univariate	17	1371/1345	57	1.53 (1.22-1.91)	< 0.001	
Multivariate	6	296/346	62.7	1.54 (0.95-2.50)	0.079	
Survival data source						0.542
Reported	12	664/697	56.7	1.63 (1.21-2.19)	0.001	
Estimated	11	1003/994	55.8	1.42 (1.09-1.87)	0.011	
Scoring method						0.133^#^
IRS or H-score	13	703/605	45	1.83 (1.40-2.38)	< 0.001	
Count, percentage, or intensity only	6	684/800	0	1.30 (1.06-1.59)	0.011	
NR	4	280/286	78.2	1.13 (0.62-2.07)	0.683	
Cutoff determination						0.478
Median values	8	620/596	63.9	1.80 (1.23-2.65)	0.003	
Fixed values	10	713/789	0	1.45 (1.22-1.73)	< 0.001	
Study quality						0.869
High	16	1263/1337	33.9	1.50 (1.23-1.83)	< 0.001	
Low or moderate	7	404/354	78.5	1.45 (0.88-2.46)	0.143	
Tumor type						NA
Breast cancer	3	204/172	51.5	0.72 (0.33-1.56)	0.399	
CRC	3	488/509	22.8	1.36 (1.02-1.82)	0.038	
Gastric cancer	2	110/94	70.7	2.32 (1.06-5.05)	0.034	
Lung cancer	3	169/271	0	1.13 (0.79-1.62)	0.501	
Osteosarcoma	2	32/56	0	2.52 (1.36-4.67)	0.003	

^#^ Comparing subgroup of IRS or H-score to subgroup of count, percentage, or intensity only.

CI, confidence interval; HR, hazard ratio; IRS, immunoreactive score; NA, not applicable.

In tumor-specific analyses, high Siglec-15 expression was significantly associated with poorer OS in CRC (HR = 1.36, 95% CI: 1.02-1.82, *P* = 0.038), gastric cancer (HR = 2.32, 95% CI: 1.06-5.05, *P* = 0.034), and osteosarcoma (HR = 2.52, 95% CI: 1.36-4.67, *P* = 0.003, **Supplementary Figure 8**). In breast cancer, there was a non-significant association in the opposite direction (HR = 0.72, 95% CI: 0.33-1.56, *P* = 0.399).

### PFS

**Figure 3:**
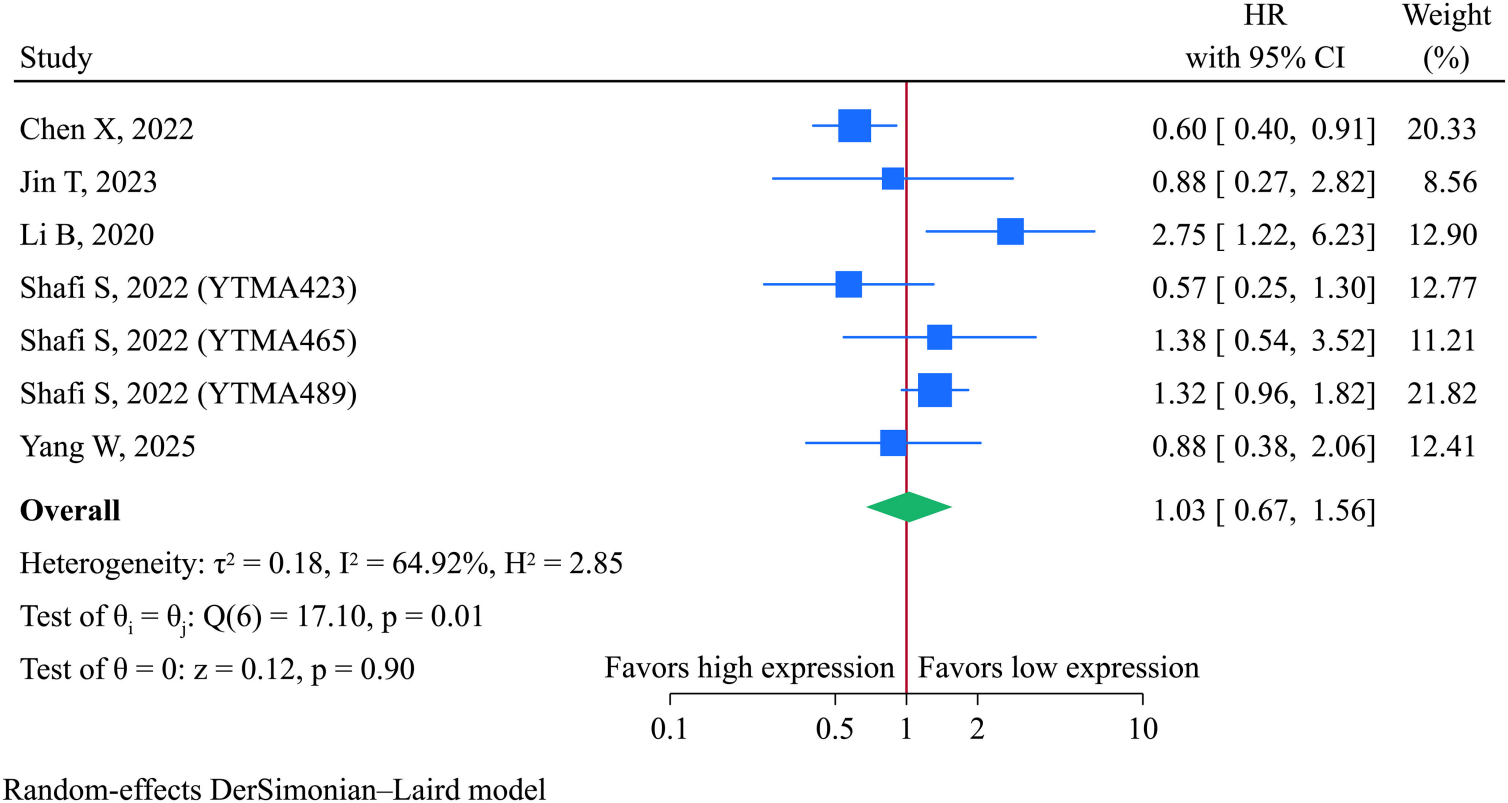
Forest plot of the association between tumoral Siglec-15 expression and PFS in solid tumors. HR, hazard ratio; PFS, progression-free survival.

Seven datasets involving 441 high-expression and 738 low-expression patients were included. No significant association was observed between Siglec-15 expression and PFS (HR = 1.03, 95% CI: 0.67-1.56, *P* = 0.903, **Figure 3**).

### DFS/RFS and DSS

**Figure 4:**
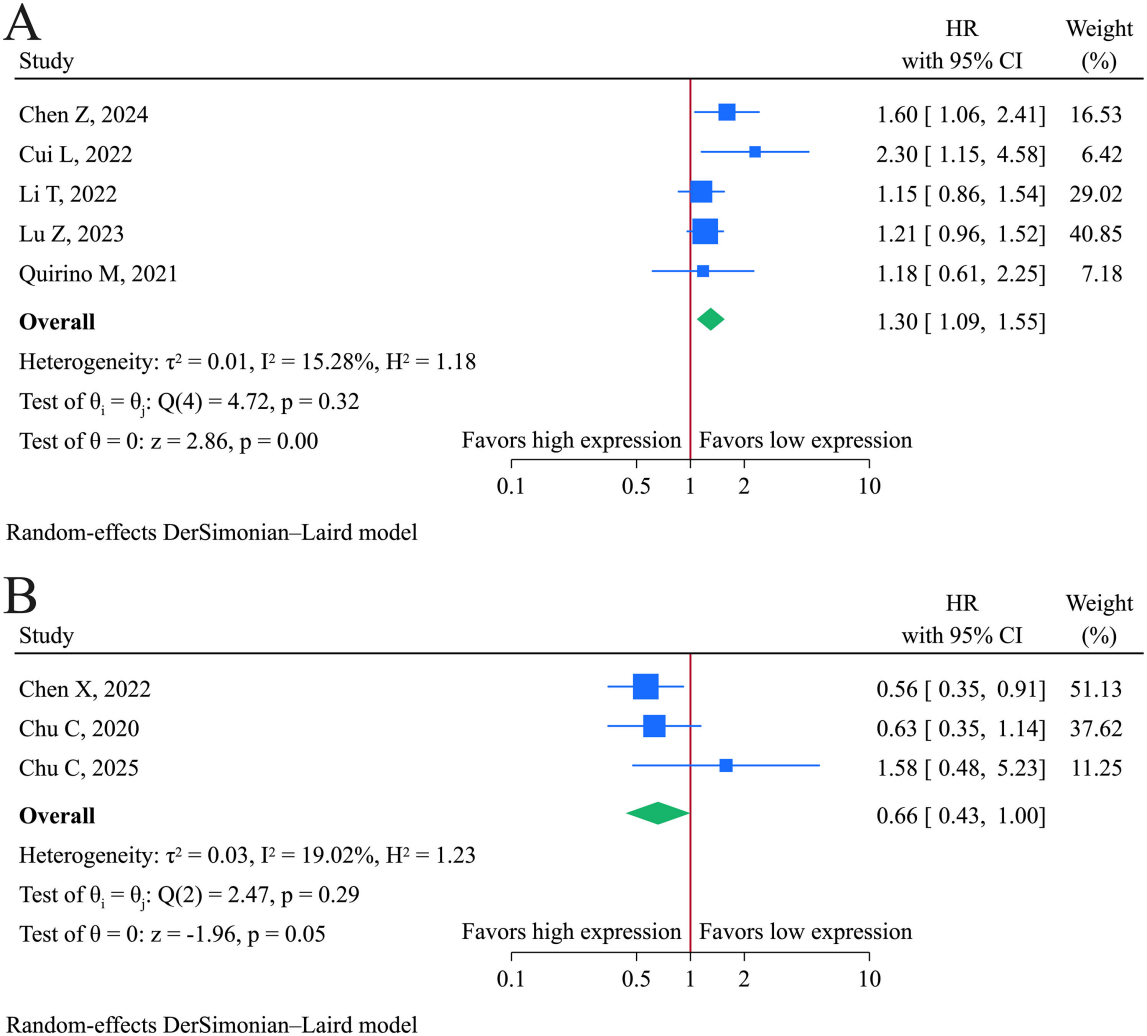
Forest plot of the association between tumoral Siglec-15 expression and DFS/RFS **(A)** and DSS **(B)** in solid tumors. DFS, disease-free survival; DSS, disease-specific survival; HR, hazard ratio; RFS, relapse-free survival.

Five studies (730 high-expression *vs*. 667 low-expression patients) showed that high Siglec-15 expression was significantly associated with poorer DFS/RFS (HR = 1.30, 95% CI: 1.09-1.55, *P* = 0.004, **Figure 4A**). In contrast, pooled analysis of three studies suggested a borderline association between high Siglec-15 expression and improved DSS (HR = 0.66, 95% CI: 0.43-1.00, *P* = 0.050, **Figure 4B**).

### Clinicopathological features

**Figure 5:**
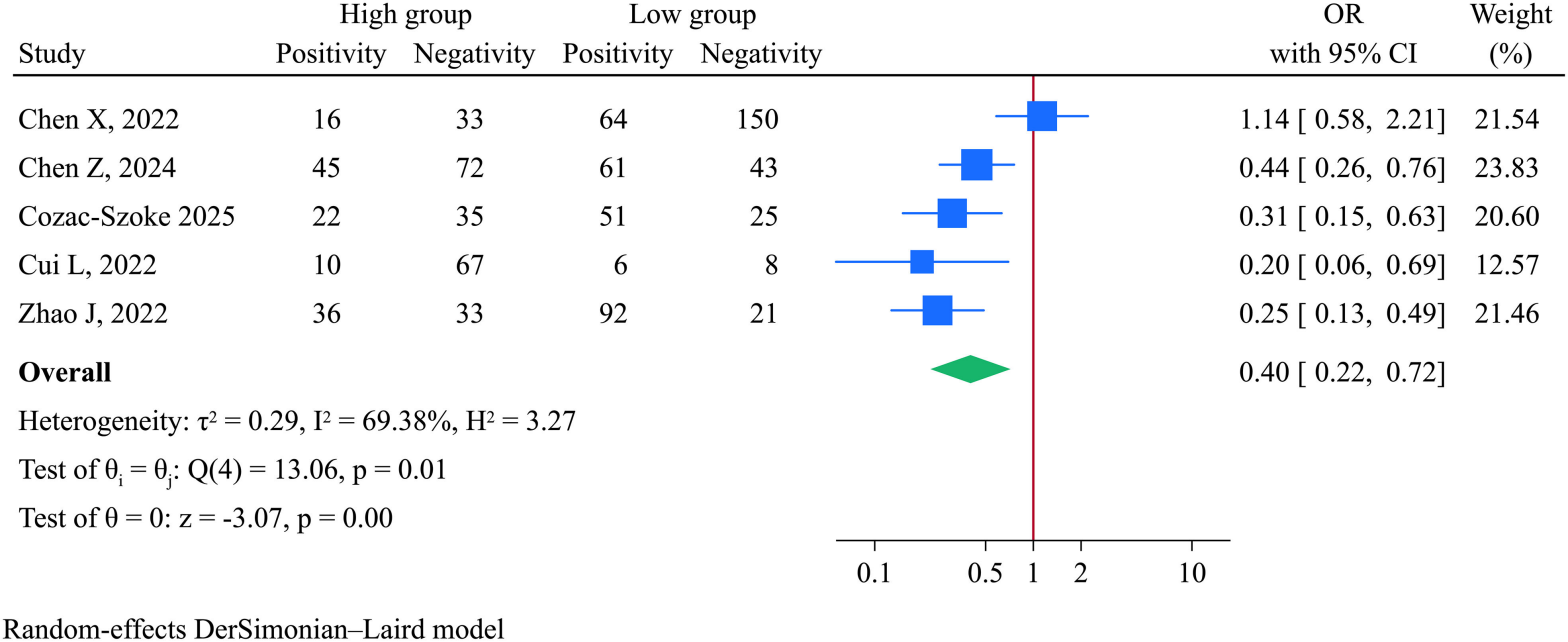
Forest plot of the association between tumoral Siglec-15 expression and PD-L1 positivity. OR, odds ratio; PD-L1, programmed death ligand-1.

Results of Siglec-15 protein expression in association with clinicopathological features are summarized in **Table 3**. No significant associations were observed between Siglec-15 expression and sex (OR = 1.05, 95% CI: 0.88-1.25, **Supplementary Figure 9**), age (OR = 1.05, 95% CI: 0.89-1.24, **Supplementary Figure 10**), tumor differentiation (OR = 0.87, 95% CI: 0.53-1.41, **Supplementary Figure 11**), tumor size (OR = 1.25, 95% CI: 0.81-1.97, **Supplementary Figure 12**), LNM (OR = 1.44, 95% CI: 0.86-2.42, **Supplementary Figure 13**), distant metastasis (OR = 1.34, 95% CI: 0.51-3.55, **Supplementary Figure 14**), or TNM stage (OR = 1.29, 95% CI: 0.95-1.75, **Supplementary Figure 15**). However, high Siglec-15 expression was significantly associated with lower PD-L1 positivity in tumor cells (OR = 0.40, 95% CI: 0.23-0.72, *P* = 0.002, **Figure 5**).

**Table 3:** Main analyses of the association between Siglec-15 expression and clinicopathological features.

Outcomes	No. of studies	Total samples	*I*^2^ (%)	OR (95% CI)	*P* for effect size
Gender (male *vs*. female)	23	3323	9.7	1.05 (0.88-1.25)	0.594
Age (older *vs*. younger)	19	2787	0	1.05 (0.89-1.24)	0.549
Tumor differentiation (poor *vs*. well/moderate)	14	2394	76	0.87 (0.53-1.41)	0.559
Tumor size (large *vs*. small)	8	997	45.6	1.26 (0.81-1.97)	0.302
LNM (yes *vs*. no)	13	1740	74.4	1.44 (0.86-2.42)	0.171
Distant metastasis (yes *vs*. no)	6	678	50.7	1.34 (0.51-3.55)	0.557
TNM stage (III/IV *vs*. I/II)	17	2894	49.8	1.29 (0.95-1.75)	0.108
PD-L1 (positivity *vs*. negativity)	5	890	69.4	0.40 (0.23-0.72)	0.002

LNM, lymph node metastasis; OR, odds ratio; PD-L1, programmed death-ligand 1.

Notably, subgroup analyses revealed that the absence of significant overall associations of clinicopathological features may be partially driven by methodological heterogeneity in cutoff definitions (**Supplementary Table 6**). When using fixed cutoff values, high Siglec-15 expression was significantly associated with increased risk of LNM (n = 5, OR = 2.10, 95% CI: 1.06-4.13, *P* = 0.033) and advanced TNM stage (n = 5, OR = 2.53, 95% CI: 1.40-4.58, *P* = 0.002). In lung cancer specifically, similar associations were observed for LNM (n = 2, OR = 1.99, 95% CI: 1.01-3.93, *P* = 0.046) and advanced stage (n = 2, OR = 1.87, 95% CI: 1.11-3.16, *P* = 0.019).

*Meta-regression, sensitivity, and publication bias*

Meta-regression analyses indicated that most baseline characteristics did not significantly influence effect sizes, except for the proportion of male patients (*P* = 0.002), where higher male proportion was associated with stronger effect estimates (**Supplementary Table 7**; **Supplementary Figure 16A**). However, the association became non-significant after excluding three breast cancer studies (*P* = 0.058, **Supplementary Figure 16B**). Leave-One-Out sensitivity analyses showed that the pooled results of outcomes were robust except DSS (**Supplementary Figure 17**). After excluding the study of Chu X et al. or Chu C et al. (23, 34), the association between Siglec-15 expression and DSS was not significant (*P* = 0.721 and 0.671, respectively). Sensitivity analysis of OS excluding all curve-estimated HRs showed that the pooled estimates remained statistically significant (HR = 1.63, 95% CI: 1.21-2.19, *P* = 0.001; **Supplementary Figure 18**), indicating that reconstructed HRs did not significantly affect the overall conclusions. Funnel plots of all outcomes were basically symmetric (**Supplementary Figure 19**), and Egger’s test of outcomes with at least 10 available studies indicated no significant publication bias (**Supplementary Table 8**).

## Discussion

In this meta-analysis including 27 studies and 4075 patients, we found that elevated tumoral Siglec-15 expression was significantly associated with poorer OS and worse DFS/RFS in patients with solid tumors. In contrast, no significant association was observed for PFS, while a marginal association with improved DSS was identified based on a limited number of studies. We additionally demonstrated an inverse relationship between tumoral Siglec-15 expression and PD-L1 positivity, supporting the concept that Siglec-15 may function as an alternative immune checkpoint pathway independent of the PD-1/PD-L1 axis. Collectively, these findings indicate that Siglec-15 has potential clinical value as both a prognostic biomarker and a therapeutic target in multiple solid malignancies.

The adverse prognostic impact associated with elevated Siglec-15 expression is consistent with accumulating evidence regarding its immunosuppressive and tumor-promoting functions (47, 48). Unlike classical immune checkpoints that primarily regulate lymphocyte activation, Siglec-15 appears to influence both immune and tumor cell compartments. Experimental studies have consistently shown that Siglec-15 contributes to the establishment of an immunosuppressive tumor microenvironment (TME) through multiple mechanisms (49). Siglec-15 is highly expressed on TAMs and promotes polarization toward an M2-like phenotype, which is characterized by enhanced secretion of immunosuppressive cytokines and reduced antitumor immune activity (20, 50). Engagement of Siglec-15 activates the DAP12-Syk signaling pathway and stimulates downstream production of transforming growth factor-β (TGF-β) and other mediators that suppress cytotoxic T-cell responses (16). As a result, tumors expressing high levels of Siglec-15 may acquire a greater capacity to evade immune surveillance and sustain malignant progression (51).

In addition to its effects on the immune microenvironment, accumulating evidence suggests that Siglec-15 also exerts tumor-intrinsic functions. Several experimental studies have demonstrated that Siglec-15 promotes proliferation, migration, invasion, and resistance to apoptosis through activation of PI3K/Akt, MAPK, STAT3, and JAK2/STAT3 signaling pathways (17–19, 52–54). These observations may explain why high Siglec-15 expression was associated with significantly shorter survival despite the absence of consistent associations with conventional clinicopathological characteristics in our pooled analyses. The biological influence of Siglec-15 may extend beyond tumor stage or nodal status and instead reflect dynamic interactions between tumor cells and the immune microenvironment that are not captured by routine pathological variables.

Our meta-analysis revealed significant association of high Siglec-15 expression with worsened OS and DFS/RFS but a borderline association with improved DSS. The apparent discrepancy between these survival outcomes likely reflects several interrelated factors. The DSS analysis was based on three studies (23, 33, 34), resulting in reduced statistical stability and wider uncertainty as shown by Leave-One-Out sensitivity analysis. Differences in tumor type distribution may also contribute to this inconsistency, as DSS data were predominantly derived from PDAC and peSCC showing “cold tumor” immunophenotype (23, 34). OS is influenced not only by cancer progression but also by non-cancer-related mortality, which may be particularly relevant in cohorts with advanced disease and older patient populations. Moreover, treatment-related factors, including variability in systemic therapies and the increasing use of immune checkpoint inhibitors, may differentially influence OS and DFS without necessarily altering cancer-specific mortality. Collectively, these factors suggest that the observed DSS association should be interpreted with caution and requires validation in larger, tumor-specific cohorts.

Notably, the prognostic effect of Siglec-15 was not uniform across cancer types. Significant associations with poor OS were observed in CRC (HR = 1.36, 95% CI: 1.02-1.82), gastric cancer (HR = 2.32, 95% CI: 1.06-5.05), and osteosarcoma (HR = 2.52, 95% CI: 1.36-4.67). These findings are consistent with previous mechanistic studies suggesting that Siglec-15 contributes to tumor progression through distinct pathways in different malignancies. In CRC, elevated Siglec-15 expression has been linked to reduced lymphocyte infiltration and an immune-excluded phenotype, both of which are associated with unfavorable clinical outcomes (22). In gastric cancer, Siglec-15 expression correlates with aggressive pathological characteristics, including poor differentiation and lymphovascular invasion (21, 26). Osteosarcoma exhibited the largest effect size among all tumor types analyzed, supporting previous evidence that Siglec-15-mediated activation of STAT3/BCL-2 signaling promotes tumor cell survival and metastatic dissemination (17, 19).

Interestingly, breast cancer demonstrated a different pattern. Although the pooled estimate did not reach statistical significance, a trend toward improved OS was observed among patients with high Siglec-15 expression (HR = 0.72, 95% CI: 0.33-1.56). Similar findings have been reported in several independent studies and bioinformatic analyses using TCGA, GTEx, and KMplotter databases (25). One possible explanation is that the biological role of Siglec-15 may depend on the underlying immune contexture of individual tumor types. In breast cancer, particularly hormone receptor-positive subtypes, Siglec-15 expression has been associated with increased immune cell infiltration and less aggressive clinicopathological characteristics (25, 27). These observations suggest that the prognostic significance of Siglec-15 is likely context dependent rather than universally detrimental across all cancers. Future studies incorporating spatial and single-cell profiling technologies may help clarify these tumor-specific differences.

A particularly important finding of our study was the significant inverse association between Siglec-15 and PD-L1 expression (OR = 0.40, 95% CI: 0.23-0.72). Since its initial description as a novel immune suppressor, Siglec-15 has attracted attention because of its largely mutually exclusive expression pattern with PD-L1 (11). Subsequent studies have confirmed that co-expression of Siglec-15 and PD-L1 occurs in only a small minority of cases, with one analysis reporting the co-expression rate as low as approximately 3% in NSCLC (20). Our pooled analysis provides quantitative evidence supporting this relationship. Mechanistically, this phenomenon may be explained by differential regulation by interferon-γ (IFN-γ). While IFN-γ strongly induces PD-L1 expression, persistent exposure suppresses Siglec-15 expression (11). Consequently, tumors may preferentially utilize either the PD-L1 pathway or the Siglec-15 pathway to achieve immune escape. Patients with double positivity of Siglec-15 and PD-L1 experienced the worse survival of any subgroup in gastric cancer and nasopharyngeal carcinoma (21, 46). This observation has important therapeutic implications. A substantial proportion of patients with PD-L1-negative tumors derive limited benefit from currently available PD-1/PD-L1 inhibitors. Siglec-15 may represent an alternative immune checkpoint that maintains immunosuppression in these tumors. Therefore, therapeutic targeting of Siglec-15 could potentially expand the population of patients eligible for immune checkpoint therapy. Early-phase clinical studies evaluating anti-Siglec-15 antibodies, including NC318 (NCT03665285) and PYX-106 (NCT05718557), have already demonstrated encouraging signals of activity, particularly in patients with tumors resistant to conventional immunotherapy. Although definitive clinical evidence remains limited, the biological rationale for Siglec-15-directed treatment continues to strengthen. The combination of anti-PD-1 and anti-Siglec-15 blockade may be particularly appropriate for patients with dual-positive tumors, while those with Siglec-15-positive/PD-L1-negative disease may be optimal candidates for anti-Siglec-15 monotherapy.

While combined blockade of PD-1/PD-L1 and Siglec-15 represents a promising therapeutic strategy, potential safety concerns must be carefully considered. Siglec-15 is physiologically expressed in osteoclasts and myeloid cells, where it plays a role in bone remodeling and immune homeostasis (12). Therefore, dual inhibition may theoretically disrupt bone metabolism and lead to adverse skeletal events such as osteoporosis, impaired bone remodeling, or increased fracture risk. In a clinical trial of NC318 (NCT03665285), pathological fracture was observed in one patient receiving NC318 at 240 mg dose. Another trial of NC318 alone or in combination with pembrolizumab in advanced NSCLC is ongoing without posting safety profiles (NCT04699123). Future, long-term studies should incorporate systematic monitoring of bone metabolism markers and skeletal outcomes to fully evaluate the safety profile of dual checkpoint blockade.

Another clinically relevant observation from our analyses concerns the substantial methodological heterogeneity among studies evaluating Siglec-15 expression. Considerable variability existed in both IHC scoring systems and cutoff value determination methods. Unlike PD-L1, for which clinically validated scoring algorithms such as the tumor proportion score (TPS) and combined positive score (CPS) have been established, no standardized approach currently exists for assessing Siglec-15 expression. This lack of standardization represents one of the major barriers to the clinical implementation of Siglec-15 as a prognostic biomarker. Interestingly, our subgroup analyses suggested that studies using fixed cutoff values exhibited remarkably consistent results, with virtually no between-study heterogeneity observed for OS (*I^2^* = 0%, n = 10). Similarly, studies applying relatively simple scoring approaches based on staining percentage, cell count, or staining intensity alone showed substantially lower heterogeneity than those using composite scoring systems such as the IRS or H-score (0% *vs.* 45%). These findings imply that methodological differences in biomarker assessment may contribute more to variability in reported outcomes than biological differences across study populations.

The issue of cutoff value selection deserves particular attention. Among the included studies, most investigators used predefined fixed thresholds (n = 12), whereas others adopted median values derived from their own cohorts (n = 9) or statistically optimized cutoffs generated using methods such as X-tile analysis (n = 2). Although optimal cutoffs may maximize prognostic discrimination within a single dataset, they may also reduce reproducibility and increase the risk of overfitting. Conversely, median-based cutoffs facilitate internal comparisons but often lack external validity because the distribution of Siglec-15 expression varies considerably across tumor types and populations. Our findings suggest that future research should prioritize the development of clinically applicable and biologically informed cutoff strategies. Similar to the evolution of PD-L1 assessment, large multicenter studies incorporating clinical outcomes and therapeutic response data will likely be required to establish universally accepted scoring criteria for Siglec-15.

An additional issue is the spatial distribution of Siglec-15 expression within the TME. The present meta-analysis specifically focused on Siglec-15 expression in tumor cells. However, accumulating evidence suggests that Siglec-15 expression on stromal cells and TAMs may have distinct, and sometimes opposite, prognostic implications compared to tumoral expression. In PDAC, for example, tumoral Siglec-15 expression was associated with favorable survival outcomes (23), whereas Siglec-15-positive TAMs were linked to poor prognosis (38). Similar discrepancies have been reported in colon adenocarcinoma and primary central nervous system lymphoma, where stromal or macrophage-associated Siglec-15 expression showed stronger prognostic significance than expression within tumor cells themselves (45, 55). Recent studies using multiplex immunofluorescence and spatial transcriptomic approaches have provided further insights into this phenomenon. In NSCLC, Siglec-15-positive macrophages accumulate predominantly within stromal regions surrounding tumor nests and form a physical and immunological barrier that restricts CD8^+^ T-cell infiltration (20). This spatial organization contributes to an immune-excluded phenotype characterized by ineffective antitumor immunity despite the presence of cytotoxic lymphocytes in the tumor microenvironment (20, 56). Such findings highlight the possibility that stromal and macrophage-associated Siglec-15 expression may represent more informative biomarkers of immune suppression than tumoral expression alone. Emerging evidence suggests that Siglec-15 expression within the TME is highly compartmentalized, and stromal or macrophage-associated expression may exert distinct or even opposite biological effects compared with tumor-cell expression. Therefore, the prognostic value of tumoral Siglec-15 alone may not fully capture its complex immunoregulatory role and could potentially lead to misclassification when used as a standalone biomarker for predicting immunotherapy response. Integrated assessment of tumoral expression, stromal expression, macrophage infiltration, and PD-L1 status may ultimately provide a more accurate framework for identifying patients most likely to benefit from Siglec-15-directed therapies.

Several strengths of this study should be acknowledged. First, this represents the largest and most comprehensive meta-analysis evaluating the prognostic significance of tumoral Siglec-15 expression in solid tumors to date, including 27 studies and 4075 patients. Compared with a previous meta-analysis including 13 studies and 1376 patients (28), the substantially larger sample size provided greater statistical power and enabled more detailed subgroup analyses. Second, we systematically evaluated multiple survival outcomes and clinicopathological characteristics, providing a comprehensive assessment of the clinical relevance of Siglec-15. Third, extensive subgroup analyses and meta-regression analyses were performed to explore potential sources of heterogeneity and assess the robustness of the findings. Finally, our study highlights several methodological and biological factors that may account for inconsistencies in the existing literature and may help guide future research in this rapidly evolving field.

Nevertheless, several limitations should also be considered when interpreting our findings. (1) Most included studies originated from China, which may limit the generalizability of the results to other ethnic and geographic populations. (2) All eligible studies were observational and retrospective in nature, making them susceptible to selection bias and residual confounding. (3) Substantial variability existed in IHC scoring systems, and cutoff definitions, which may have influenced pooled estimates despite our subgroup analyses. (4) The number of studies available for several tumor types and survival outcomes remained limited, reducing the precision of cancer-specific estimates. (5) Because our analyses focused exclusively on tumor-cell expression, the prognostic significance of stromal and macrophage-associated Siglec-15 could not be quantitatively evaluated. (6) The median follow-up duration varied substantially across the included studies. Since OS is inherently time-dependent, differences in follow-up length may have influenced the maturity of survival events and contributed to between-study variability. Although HRs partially account for differences in follow-up through time-to-event analyses, residual heterogeneity related to follow-up duration cannot be completely excluded. (7) Most studies only provided univariate HRs that are susceptible to clinicopathological confounders. Subgroup analysis showed nearly identical pooled estimates between univariable and multivariable analyses, suggesting that the overall findings are robust. However, the subgroup of multivariate analysis showed wider confidence interval (0.95-2.50), implying the need of more studies. (8) Only univariable meta-regression analyses were performed owing to the limited number of studies, and multivariate meta-regression analyses exploring potential interactions between study-level covariates could not be conducted. Therefore, residual confounding among study-level covariates cannot be completely excluded. (9) Although studies based exclusively on public databases were excluded to improve pathological consistency across included cohorts, this approach may have omitted large-scale multi-omic validation datasets and thereby limited the external generalizability of our findings. (10) Dose-response meta-analysis could not be performed because virtually all included studies dichotomized Siglec-15 expression into high and low categories without reporting category-specific HRs or original continuous IHC scores.

## Conclusion

In conclusion, elevated tumoral Siglec-15 expression is associated with adverse survival outcomes in several solid tumors, particularly with respect to OS and DFS/RFS. However, its prognostic impact is highly tumor-type dependent, and a borderline inverse association with DFS was also observed. These findings highlight Siglec-15 as a context-dependent biomarker rather than a universally applicable prognostic indicator. Further large-scale, tumor-specific prospective studies are required to clarify its biological and clinical relevance and to optimize its potential as a therapeutic target in combination immunotherapy strategies.

## Data Availability

The original contributions presented in the study are included in the article/**Supplementary Material**. Further inquiries can be directed to the corresponding authors.
