# Supplementary material for: Prognostic significance of tumoral Siglec-15 expression in solid tumors: a systematic review and meta-analysis
Source: Front Immunol. 2026 Jul 21;17:1905631. doi: 10.3389/fimmu.2026.1905631 (PMC13433752; doi:10.3389/fimmu.2026.1905631)
Supplement: Supplementary file 2 [file Table2.docx]

Supplementary Table 1. Literature search strategy.

| Literature databases | Search terms | No. of articles |
| --- | --- | --- |
| PubMed | ("Siglec-15"[Title/Abstract] OR "siglec15"[Title/Abstract] OR "sialic acid-binding immunoglobulin-like lectin 15"[Title/Abstract]) AND ("Neoplasms"[Mesh] OR cancer[Title/Abstract] OR tumor[Title/Abstract] OR tumour[Title/Abstract] OR carcinoma[Title/Abstract] OR neoplasm[Title/Abstract] OR malignancy[Title/Abstract]) | 163 |
| EMBASE | ('siglec-15':ti,ab,kw OR 'siglec15':ti,ab,kw OR 'sialic acid binding immunoglobulin like lectin 15':ti,ab,kw) AND ('cancer'/exp OR 'neoplasm'/exp OR cancer:ti,ab,kw OR tumor:ti,ab,kw OR tumour:ti,ab,kw OR carcinoma:ti,ab,kw) | 224 |
| Web of Science | TS=(("Siglec-15" OR "SIGLEC15" OR "Siglec15" OR "sialic acid-binding immunoglobulin-like lectin 15") AND (cancer OR tumor OR tumour OR carcinoma OR neoplasm OR malignancy) | 248 |

Supplementary Table 2. HR estimates extracted from Kaplan-Meier plots by two extractors.

| Study | Outcome | HR (95% CI) | |
| --- | --- | --- | --- |
|  |  | Extractor 1 | Extractor 2 |
| Quirino 2021 | OS | 1.52 (0.78-2.94) | 1.43 (0.76-2.69) |
| Quirino 2021 | RFS | 1.18 (0.62-2.25) | 1.25 (0.62-2.53) |
| Fan 2021 | OS | 2.60 (0.98-6.85) | 2.38 (0.99-5.69) |
| Yang 2021 | OS | 3.22 (1.42-7.32) | 2.88 (1.34-6.20) |
| Li 2022 | OS | 1.20 (0.90-1.58) | 1.38 (0.89-2.13) |
| Li 2022 | DFS | 1.15 (0.86-1.54) | 1.23 (0.87-1.73) |
| Song 2022 | OS | 2.47 (1.11-5.50) | 2.28 (1.09-4.76) |
| Shafi 2022 (YTMA423) | OS | 0.93 (0.53-1.61) | 0.98 (0.57-1.69) |
| Shafi 2022 (YTMA423) | PFS | 0.57 (0.25-1.30) | 0.53 (0.24-1.18) |
| Shafi 2022 (YTMA489) | OS | 1.29 (0.63-2.62) | 1.20 (0.65-2.23) |
| Shafi 2022 (YTMA489) | PFS | 1.32 (0.96-1.83) | 1.39 (0.93-2.09) |
| Shafi 2022 (YTMA465) | OS | 1.26 (0.54-2.94) | 1.37 (0.59-3.16) |
| Shafi 2022 (YTMA465) | PFS | 1.38 (0.54-3.52) | 1.30 (0.53-3.19) |
| Hou 2022 | OS | 3.94 (1.46-10.64) | 3.58 (1.43-8.97) |
| Lu 2023 | DFS | 1.21 (0.96-1.52) | 1.26 (0.94-1.69) |
| Lu 2023 | OS | 1.28 (0.99-1.65) | 1.20 (1.10-1.43) |
| Chu 2025 | DSS | 1.58 (0.48-5.23) | 1.70 (0.49-5.91) |
| Yang 2025 | PFS | 0.88 (0.38-2.06) | 0.83 (0.38-1.82) |
| Tan 2025 | OS | 0.44 (0.20-0.99) | 0.42 (0.18-0.97) |

CI: confidence interval; DFS: disease-free survival; HR: hazard ratio; OS: overall survival; PFS: progression-free survival; RFS: recurrence-free survival

Supplementary Table 3. Reasons for excluding articles after full-text reviewing.

| No. | Author | Article | DOI | Excluding reasons |
| --- | --- | --- | --- | --- |
| 1 | Cao X, 2022 | Identification and characterization of three Siglec15-related immune and prognostic subtypes of breast-invasive cancer | 10.1016/j.intimp.2022.108561 | Public database analysis |
| 2 | Chen Q, 2023 | Dynamic change in Siglec-15 expression in peritumoral macrophages confers an immunosuppressive microenvironment and poor outcome in glioma | 10.3389/fimmu.2023.1159085 | Data not available |
| 3 | Fudaba H, 2021 | Sialic acid-binding immunoglobulin-like lectin-15 expression on peritumoral macrophages is a favorable prognostic factor for primary central nervous system lymphoma patients | 10.1038/s41598-020-79742-9 | Hematological malignancy |
| 4 | Hao J, 2020 | The significance of Siglec-15 expression in resectable non-small cell lung cancer | 10.4149/neo_2020_200220N161 | Data not available |
| 5 | Huang Z, 2024 | Siglec-15 on macrophages suppress the immune microenvironment in patients with PD-L1 negative non-metastasis lung adenocarcinoma | 10.1038/s41417-023-00713-z | Macrophage expression |
| 6 | Li Q, 2020 | Integrative Analysis of Siglec-15 mRNA in Human Cancers Based on Data Mining | 10.7150/jca.38747 | Public database analysis |
| 7 | Li S, 2022 | Study on the effect and mechanisms of SIGLEC‑15 on laryngeal squamous cell carcinoma | 10.3760/cma.j.cn115330-20210705-00424 | Chinese language |
| 8 | Liu C, 2022 | Bioinformatics analysis of prognosis and immune microenvironment of immunological cell death-related gemcitabine-resistance genes in bladder cancer | 10.21037/tau-22-736 | Public database analysis |
| 9 | McDonad K, 2021 | Development of KAM score to predict metastasis and worse survival in breast cancer | https://pmc.ncbi.nlm.nih.gov/articles/PMC8640803/ | Public database analysis |
| 10 | Qi L, 2024 | Identification of therapeutic targets and prognostic biomarkers in the Siglec family of genes in tumor immune microenvironment of sarcoma | 10.1038/s41598-023-50758-1 | Public database analysis |
| 11 | Shen L, 2025 | Bispecific Siglec-15/T cell antibody (STAB) activates T cells and suppresses pancreatic ductal adenocarcinoma and non-small cell lung tumors in vivo | 10.7150/thno.103372 | Public database analysis |
| 12 | Wang M, 2024 | The expression of Siglec-15 in epithelial ovarian cancer and its relationship with prognosis | 10.13390/j.issn.1672-1861.2024.04.003 | Chinese language |
| 13 | Zhao A, 2023 | Mechanism of PD-L1 and Siglec-15 in regulating malignant biological behavior of ovarian cancer cells and its clinical significance | 10.3872/j.issn.1007-385x.2023.02.007 | Chinese language |
| 14 | Zhao A, 2025 | Predicting ovarian cancer prognosis and immunotherapy response through siglec15 and PD-L1 expression analysis | 10.1016/j.tranon.2025.102563 | Data not available |
| 15 | Zhou S, 2023 | Association of Sialic Acid-Binding Immunoglobulin-Like Lectin 15 With Phenotypes in Esophageal Squamous Cell Carcinoma in the Setting of Neoadjuvant Chemoradiotherapy | 10.1001/jamanetworkopen.2022.50965 | Macrophage expression |

Supplementary Table 4. Clinicopathological features of studies in the meta-analysis.

| Study | Male% | TNM stage | Tumor differentiation | Clinicopathological parameters for meta-analysis | | | | | | | |
| --- | --- | --- | --- | --- | --- | --- | --- | --- | --- | --- | --- |
|  |  |  |  | Gender | Age, years | Tumor differentiation | TNM stage | Tumor size, cm | LNM | Distant metastasis | PD-L1 positivity |
| Chen X, 2022 | 54 | I/II: 209, III/IV: 54 | Poor: 92, well/moderate: 171 | yes | ≥60 vs. < 60 | yes | yes | ≥3 vs. <3 | yes |  | yes |
| Chen X, 2024 | 95.5 | I: 31, II: 25, III: 28, IV: 27 | Poor: 4, moderate: 54, well: 53 | yes | >60 vs. ≤60 | yes | yes |  | yes |  |  |
| Chen Z, 2024 | 82.4 | I: 154, II: 65, III: 2 | Poor: 60, moderate: 147, well: 8 | yes |  | yes | yes |  | yes |  | yes |
| Chu C, 2020 | 100 |  | Poor: 14, moderate: 53, well: 106 |  |  |  |  |  |  |  |  |
| Chu C, 2025 | 78.3 |  |  | yes | ≥65 vs. <65 |  |  |  | yes |  |  |
| Cozac-Szoke 2025 | 73.6 |  | Poor: 70, moderate/well: 63 | yes | ≥68 vs. <68 | yes |  | ≥5 vs. <5 | yes | yes | yes |
| Cui L, 2022 | 57.1 |  |  | yes | >60 vs. ≤60 |  |  |  |  |  | yes |
| Fan M, 2021 | 52.8 |  | Poor: 15, moderate: 11, well: 10 | yes | >20 vs. ≤20 | yes |  | ≥5 vs. <5 |  |  |  |
| Hou X, 2022 | 44.2 |  |  | yes | ≥65 vs. <65 |  |  | >2 vs. ≤2 | yes | yes |  |
| Jin T, 2023 | 25 | I: 211, II: 28, III: 4, IV: 1 |  | yes | ≥55 vs. <55 |  |  | >4 vs. ≤4 | yes | yes |  |
| Lai H, 2024 | 0 | I: 1, II: 60, III: 13 | Poor: 11, moderate: 49, well: 14 |  | ≥45 vs. <45 | yes | yes |  | yes |  |  |
| Li B, 2020 | 40.8 | I: 48, II: 18, III 37 |  | yes | >60 vs. ≤60 |  | yes |  | yes |  |  |
| Li T, 2022 | 59.3 | I: 71, II: 80, III: 58 | Poor: 38, moderate: 142, well: 28 |  |  |  |  |  |  |  |  |
| Liang G, 2025 | 63.3 | I/II: 52, III/IV: 38 | Poor: 11, moderate: 76, well: 1 | yes | ≥60 vs. < 60 | yes | yes | ≥5 vs. <5 | yes | yes |  |
| Lu Z, 2023 | 55.4 | I: 101, II: 241, III: 319, IV: 144 | Poor: 69, well/moderate: 736 | yes | ≥60 vs. < 60 | yes | yes |  |  |  |  |
| Quirino M, 2021 | 66.2 | I: 6, II: 12, III: 51, IV: 2 | Poor: 36, well/moderate: 35 | yes | ≥60 vs. < 60 | yes | yes |  | yes |  |  |
| Shafi S, 2022 (YTMA423) | 39.4 | I: 197, II: 36, III: 12, IV: 3 |  | yes |  |  | yes |  |  |  |  |
| Shafi S, 2022 (YTMA465) | 70 | I: 2, II: 8, III: 5, IV: 49 | Poor: 29, moderate: 32, well: 2 | yes |  | yes | yes |  |  |  |  |
| Shafi S, 2022 (YTMA489) | 1.2 | I: 81, II: 95, III: 37, IV: 3 | Poor: 72, moderate: 105, well: 31 |  |  | yes | yes |  |  |  |  |
| Shafi S, 2022 (YTMA497) | 46.9 | I: 6, II:2, III: 18, IV: 6 |  | yes |  |  | yes |  |  |  |  |
| Shafi S, 2022 (YTMA507) | 86.5 |  |  | yes |  |  |  |  |  |  |  |
| Song K, 2022 | 63.5 |  |  | yes | >25 vs. ≤25 |  |  | >5 vs ≤5 |  |  |  |
| Sun H, 2024 | 53.8 | I/II: 45, III/IV: 44 | Poor: 27, well/moderate: 66 | yes | ≥60 vs. < 60 | yes | yes | ≥3 vs. <3 | yes | yes |  |
| Tan Z, 2025 | 0 |  |  |  |  |  |  |  |  |  |  |
| Wang J, 2023 | 50 |  |  |  |  |  |  |  |  |  |  |
| Yang W, 2021 (cohort 1) | 71.3 | I/II: 138, III/IV: 12 | Poor: 47, well/moderate: 103 | yes | >65 vs. ≤65 | yes | yes |  |  |  |  |
| Yang W, 2021 (cohort 2) | 78.1 | I/II: 17, III/IV: 15 | Poor: 9, well/moderate: 23 | yes | >65 vs. ≤65 | yes | yes |  |  | yes |  |
| Yang W, 2025 | 72.5 | III: 2, IV: 38 |  |  |  |  |  |  |  |  |  |
| Zhan W, 2023 | 56.9 | I/II: 63, III/IV: 39 |  | yes | >68 vs. ≤68 |  | yes |  |  |  |  |
| Zhao J, 2022 | 70.3 | I/II: 48, III/IV: 134 |  | yes | >48 vs. ≤48 |  | yes |  | yes |  | yes |

LNM: lymph node metastasis.

Supplementary Table 5. Quality assessment using Newcastle-Ottawa Scale for cohort studies.

| Study | 1. Selection | | | | 2. Comparability | 3. Outcome | | | Total |
| --- | --- | --- | --- | --- | --- | --- | --- | --- | --- |
|  | 1.1 Representativeness of the exposed cohort ^a^ | 1.2 Selection of the non-exposed cohort | 1.3 Ascertainment of exposure ^b^ | 1.4 Outcome not present at the start of study | Comparability of cohorts on the basis of the design or analysis ^c^ | 3.1 Assessment of outcome | 3.2 Was follow-up long enough for outcomes to occur | 3.3 Adequacy of follow-up of cohorts |  |
| Chen X, 2022 | * | * | - | * | * | * | * | * | 7 |
| Chen X, 2024 | * | * | - | * | * | * | * | * | 8 |
| Chen Z, 2024 | * | * | - | * | * | * | * | * | 8 |
| Chu C, 2020 | * | * | * | * | - | * | * | * | 7 |
| Chu C, 2025 | * | * | * | * | ** | * | * | * | 9 |
| Cozac-Szoke 2025 | * | * | - | * | - | * | * | * | 6 |
| Cui L, 2022 | * | * | * | * | * | * | * | * | 8 |
| Fan M, 2021 | * | * | * | * | * | * | * | * | 8 |
| Hou X, 2022 | * | * | - | * | - | * | * | * | 6 |
| Jin T, 2023 | * | * | - | * | * | * | * | * | 7 |
| Lai H, 2024 | - | * | - | * | * | * | * | * | 6 |
| Li B, 2020 | * | * | * | * | ** | * | * | * | 9 |
| Li T, 2022 | * | * | - | * | - | * | * | * | 6 |
| Liang G, 2025 | - | * | * | * | - | * | * | * | 6 |
| Lu Z, 2023 | * | * | * | * | * | * | * | * | 8 |
| Quirino M, 2021 | * | * | * | * | - | * | * | * | 7 |
| Shafi S, 2022 | * | * | - | * | ** | * | * | * | 8 |
| Song K, 2022 | * | * | * | * | * | * | * | * | 8 |
| Sun H, 2024 | - | * | * | * | * | * | * | * | 7 |
| Tan Z, 2025 | * | * | - | * | - | * | * | * | 6 |
| Wang J, 2023 | * | * | * | * | - | * | * | * | 7 |
| Yang W, 2021 | - | * | - | * | * | * | * | * | 7 |
| Yang W, 2025 | * | * | * | * | - | * | * | * | 7 |
| Zhan W, 2023 | - | * | - | * | * | * | * | * | 6 |
| Zhao J, 2022 | * | * | - | * | ** | * | * | * | 8 |

^a^ Studies collecting samples from hospitals were given one star.

^b^ Studies defining high/low Siglec-15 expression using fixed cutoff values were given one star.

^c^ Controlled for clinicopathological features.

Supplementary Table 6. Subgroup analyses of the association between Siglec-15 expression and clinicopathological features.

| Subgroup | No. of studies | Total samples | *I*^2^ (%) | OR (95% CI) | *P* for effect size | *P* for subgroup difference |
| --- | --- | --- | --- | --- | --- | --- |
| **Gender (male *vs*. female)** |  |  |  |  |  |  |
| Country |  |  |  |  |  | 0.874 |
| China | 17 | 2730 | 30.2 | 1.06 (0.84-1.34) | 0.622 |  |
| Outside China | 6 | 593 | 0 | 1.08 (0.74-1.57) | 0.682 |  |
| Sample size |  |  |  |  |  | 0.664 |
| < 100 | 12 | 759 | 0 | 1.12 (0.81-1.55) | 0.489 |  |
| ≥ 100 | 11 | 2564 | 33.8 | 1.04 (0.81-1.33) | 0.768 |  |
| Scoring method |  |  |  |  |  | 0.200^#^ |
| IRS or H-score | 13 | 1418 | 21.3 | 1.13 (0.86-1.50) | 0.378 |  |
| Count, percentage, or intensity only | 8 | 1612 | 0 | 0.91 (0.74-1.13) | 0.396 |  |
| NR | 2 | 293 | 59.5 | 1.01 (0.18-5.74) | 0.988 |  |
| Cutoff determination |  |  |  |  |  | 0.377 |
| Median values | 11 | 1357 | 0 | 1.12 (0.87-1.43) | 0.392 |  |
| Fixed values | 9 | 1410 | 14.6 | 0.98 (0.73-1.31) | 0.884 |  |
| Tumor type |  |  |  |  |  | NA |
| Bladder cancer | 3 | 138 | 37.6 | 1.54 (0.40-5.98) | 0.534 |  |
| CRC | 3 | 997 | 0 | 0.91 (0.70-1.17) | 0.448 |  |
| Gastric cancer | 2 | 204 | 0 | 1.10 (0.58-2.09) | 0.762 |  |
| Lung cancer | 3 | 446 | 0 | 0.97 (0.63-1.49) | 0.874 |  |
| Thyroid cancer | 2 | 330 | 0 | 0.71 (0.43-1.17) | 0.181 |  |
| ccRCC | 2 | 182 | 0 | 1.64 (0.85-3.18) | 0.143 |  |
| Osteosarcoma | 2 | 88 | 58.4 | 0.69 (0.16-2.90) | 0.614 |  |
| **Age (older *vs*. younger)** |  |  |  |  |  |  |
| Country |  |  |  |  |  | 0.669 |
| China | 17 | 2583 | 0 | 1.04 (0.88-1.24) | 0.648 |  |
| Outside China | 2 | 204 | 61.6 | 1.38 (0.49-3.93) | 0.543 |  |
| Sample size |  |  |  |  |  | 0.204 |
| <100 | 10 | 694 | 0 | 0.87 (0.63-1.22) | 0.418 |  |
| ≥100 | 9 | 2093 | 0 | 1.12 (0.93-1.35) | 0.250 |  |
| Scoring method |  |  |  |  |  | 0.265^#^ |
| IRS or H-score | 13 | 1271 | 0 | 0.95 (0.73-1.22) | 0.663 |  |
| Count, percentage, or intensity only | 4 | 1223 | 0 | 1.15 (0.91-1.47) | 0.247 |  |
| NR | 2 | 293 | 0 | 1.07 (0.67-1.71) | 0.780 |  |
| Cutoff determination |  |  |  |  |  | 0.557^&^ |
| Median values | 6 | 747 | 0 | 0.96 (0.69-1.35) | 0.813 |  |
| Fixed values | 9 | 1410 | 5 | 1.06 (0.82-1.36) | 0.677 |  |
| Optimal values | 2 | 337 | 0 | 1.09 (0.63-1.87) | 0.758 |  |
| Age cutoff value |  |  |  |  |  | 0.417 |
| 60 years | 8 | 1627 | 0 | 1.15 (0.93-1.42) | 0.193 |  |
| 65 years | 4 | 337 | 0 | 0.91 (0.55-1.53) | 0.730 |  |
| Tumor type |  |  |  |  |  | NA |
| CRC | 3 | 997 | 28.9 | 1.02 (0.68-1.52) | 0.939 |  |
| Gastric cancer | 2 | 204 | 61.6 | 1.38 (0.49-3.93) | 0.543 |  |
| Lung cancer | 2 | 195 | 0 | 0.80 (0.41-1.59) | 0.529 |  |
| Thyroid cancer | 2 | 330 | 0 | 0.91 (0.53-1.57) | 0.729 |  |
| ccRCC | 2 | 182 | 0 | 1.21 (0.58-2.52) | 0.616 |  |
| **Tumor differentiation (poor *vs*. well/moderate)** |  |  |  |  |  |  |
| Country |  |  |  |  |  | 0.964 |
| China | 10 | 1875 | 71.8 | 0.86 (0.49-1.51) | 0.600 |  |
| Outside China | 4 | 519 | 84.8 | 0.84 (0.29-2.42) | 0.741 |  |
| Sample size |  |  |  |  |  | 0.069 |
| < 100 | 7 | 466 | 64.6 | 0.52 (0.24-1.14) | 0.103 |  |
| ≥ 100 | 7 | 1928 | 79.6 | 1.27 (0.70-2.28) | 0.430 |  |
| Scoring method |  |  |  |  |  | 0.930^#^ |
| IRS or H-score | 9 | 990 | 78.6 | 0.86 (0.42-1.75) | 0.667 |  |
| Count, percentage, or intensity only | 4 | 1383 | 81.5 | 0.84 (0.39-1.81) | 0.653 |  |
| NR | 1 | 111 |  |  |  |  |
| Cutoff determination |  |  |  |  |  | 0.188^&^ |
| Median values | 6 | 851 | 75.8 | 1.42 (0.75-2.69) | 0.282 |  |
| Fixed values | 5 | 1095 | 66.4 | 0.72 (0.33-1.58) | 0.412 |  |
| Optimal values | 2 | 337 | 39.2 | 0.29 (0.11-0.75) | 0.011 |  |
| Tumor type |  |  |  |  |  | NA |
| Breast cancer | 2 | 319 | 92.4 | 0.63 (0.04-9.26) | 0.737 |  |
| Gastric cancer | 2 | 204 | 91.4 | 0.53 (0.04-3.75) | 0.630 |  |
| CRC | 2 | 895 | 0 | 0.88 (0.56-1.40) | 0.596 |  |
| ccRCC | 2 | 182 | 75.3 | 2.03 (0.37-11.24) | 0.417 |  |
| **LNM (yes *vs*. no)** |  |  |  |  |  |  |
| Country |  |  |  |  |  | 0.260 |
| China | 11 | 1536 | 74 | 1.27 (0.73-2.22) | 0.403 |  |
| Outside China | 2 | 204 | 42 | 2.87 (1.13-7.29) | 0.027 |  |
| Sample size |  |  |  |  |  | 0.849 |
| < 100 | 6 | 483 | 65.8 | 1.37 (0.67-2.83) | 0.392 |  |
| ≥ 100 | 7 | 1257 | 80.9 | 1.52 (0.70-3.32) | 0.291 |  |
| Scoring method |  |  |  |  |  | 0.558^#^ |
| IRS or H-score | 9 | 1081 | 80.8 | 1.34 (0.62-2.87) | 0.456 |  |
| Count, percentage, or intensity only | 2 | 293 | 0 | 2.07 (1.16-3.70) | 0.014 |  |
| NR | 2 | 366 | 0 | 1.38 (0.74-2.56) | 0.316 |  |
| Cutoff determination |  |  |  |  |  | NA |
| Median values | 4 | 684 | 86.3 | 1.15 (0.30-4.43) | 0.842 |  |
| Fixed values | 5 | 426 | 50.7 | 2.10 (1.06-4.13) | 0.033 |  |
| Optimal values | 2 | 337 | 64.4 | 1.22 (0.43-3.45) | 0.707 |  |
| Tumor type |  |  |  |  |  | NA |
| Gastric cancer | 2 | 204 | 42 | 2.87 (1.13-7.29) | 0.027 |  |
| Lung cancer | 2 | 196 | 0 | 1.99 (1.01-3.93) | 0.046 |  |
| Thyroid cancer | 2 | 330 | 44.1 | 0.47 (0.24-0.94) | 0.032 |  |
| **TNM stage (III/IV *vs*. I/II)** |  |  |  |  |  |  |
| Country |  |  |  |  |  | 0.812 |
| China | 12 | 2226 | 57.9 | 1.32 (0.90-1.94) | 0.162 |  |
| Outside China | 5 | 758 | 23 | 1.17 (0.70-1.95) | 0.541 |  |
| Sample size |  |  |  |  |  | 0.029 |
| < 100 | 7 | 462 | 50.1 | 1.98 (1.06-3.73) | 0.033 |  |
| ≥ 100 | 10 | 2432 | 32 | 1.06 (0.79-1.42) | 0.702 |  |
| Scoring method |  |  |  |  |  | 0.107^#^ |
| IRS or H-score | 8 | 833 | 53.8 | 1.82 (0.99-3.33) | 0.055 |  |
| Count, percentage, or intensity only | 7 | 1768 | 17.3 | 1.15 (0.85-1.56) | 0.353 |  |
| NR | 2 | 293 | 65.3 | 0.85 (0.36-2.03) | 0.716 |  |
| Cutoff determination |  |  |  |  |  | 0.016^&^ |
| Median values | 8 | 1102 | 0 | 1.04 (0.72-1.50) | 0.831 |  |
| Fixed values | 5 | 1162 | 63.7 | 2.53 (1.40-4.58) | 0.002 |  |
| Optimal values | 2 | 337 | 0 | 0.57 (0.28-1.16) | 0.120 |  |
| Tumor type |  |  |  |  |  | NA |
| Breast cancer | 2 | 319 | 0 | 0.67 (0.37-1.23) | 0.201 |  |
| CRC | 3 | 997 | 77.9 | 1.92 (0.84-4.34) | 0.120 |  |
| Lung cancer | 3 | 446 | 0 | 1.87 (1.11-3.16) | 0.019 |  |
| ccRCC | 2 | 182 | 0 | 1.76 (0.71-4.38) | 0.225 |  |

^#^Comparing subgroup of IRS or H-score to subgroup of count, percentage, or intensity only.

^&^Comparing subgroup of median values to subgroup of fixed values.

ccRCC: clear cell renal cell carcinoma; CRC: colorectal cancer; IRS: immunoreactive score; LNM: lymph node metastasis; NA: not applicable; NR: not reported; OR: odds ratio.

Supplementary Table 7. Meta-regression analysis of OS in association with clinicopathological parameters.

| Parameters | No. of studies | Coefficient | Standard error | *z* value | *P* |
| --- | --- | --- | --- | --- | --- |
| Total samples | 23 | -0.0004 | 0.0006 | -0.66 | 0.506 |
| Percentage of males, % | 23 | 0.0131 | 0.0042 | 3.09 | 0.002 |
| Percentage of males excluding BC, % | 20 | 0.0138 | 0.0069 | 1.90 | 0.058 |
| Percentage of TNM III/IV stage, % | 15 | -0.0029 | 0.0040 | -0.72 | 0.470 |
| Percentage of LNM, % | 13 | -0.0064 | 0.0058 | -1.11 | 0.266 |
| Percentage of poor tumor differentiation, % | 13 | 0.0075 | 0.0080 | 0.94 | 0.347 |

BC: breast cancer; LNM: lymph node metastasis; OS: overall survival.

Supplementary Table 8. Results of Egger’s test.

| Outcomes | No. of studies | *z* value | *P* |
| --- | --- | --- | --- |
| OS | 23 | 0.95 | 0.341 |
| Gender | 23 | 0.16 | 0.875 |
| Age | 19 | -1.22 | 0.222 |
| Tumor differentiation | 14 | -1.81 | 0.071 |
| LNM | 13 | 0.45 | 0.656 |
| TNM stage | 17 | -0.29 | 0.769 |

LNM: lymph node metastasis; OS: overall survival.
