# Supplementary material for: Prognostic significance of tumoral Siglec-15 expression in solid tumors: a systematic review and meta-analysis
Source: Front Immunol. 2026 Jul 21;17:1905631. doi: 10.3389/fimmu.2026.1905631 (PMC13433752; doi:10.3389/fimmu.2026.1905631)
Supplement: Supplementary file 3 [file Table3.docx]

Supplementary Figure 1. Subgroup analysis of OS according to country.

Supplementary Figure 2. Subgroup analysis of OS according to sample size.

Supplementary Figure 3. Subgroup analysis of OS according to HR type.

Supplementary Figure 4. Subgroup analysis of OS according to survival data source.

Supplementary Figure 5. Subgroup analysis of OS according to scoring method.

Supplementary Figure 6. Subgroup analysis of OS according to cutoff determination method.

Supplementary Figure 7. Subgroup analysis of OS according to study quality.

Supplementary Figure 8. Subgroup analysis of OS according to tumor type.

Supplementary Figure 9. Forest plot of Siglec-15 expression in association with gender.

Supplementary Figure 10. Forest plot of Siglec-15 expression in association with age.

Supplementary Figure 11. Forest plot of Siglec-15 expression in association with tumor differentiation.

Supplementary Figure 12. Forest plot of Siglec-15 expression in association with tumor size.

Supplementary Figure 13. Forest plot of Siglec-15 expression in association with lymph node metastasis.

Supplementary Figure 14. Forest plot of Siglec-15 expression in association with distant metastasis.

Supplementary Figure 15. Forest plot of Siglec-15 expression in association with TNM stage.

Supplementary Figure 16. Meta-regression analysis of male percentages for OS in all studies (A) and in studies excluding breast cancer (B).

Supplementary Figure 17. Leave-One-Out sensitivity analyses of overall survival (A), progression-free survival (B), disease-free/recurrence-free survival (C), disease-specific survival (D), and PD-L1 positivity (E).

Supplementary Figure 18. Sensitivity analysis of overall survival excluding hazard ratios extracted from survival plots.

Supplementary Figure 19. Funnel plots of meta-analyses.
